# Negative Hyperselection of Patients With *RAS* and *BRAF* Wild-Type Metastatic Colorectal Cancer Who Received Panitumumab-Based Maintenance Therapy

**DOI:** 10.1200/JCO.19.01254

**Published:** 2019-09-20

**Authors:** Federica Morano, Salvatore Corallo, Sara Lonardi, Alessandra Raimondi, Chiara Cremolini, Lorenza Rimassa, Roberto Murialdo, Alberto Zaniboni, Andrea Sartore-Bianchi, Gianluca Tomasello, Patrizia Racca, Matteo Clavarezza, Vincenzo Adamo, Federica Perrone, Annunziata Gloghini, Elena Tamborini, Adele Busico, Antonia Martinetti, Federica Palermo, Fotios Loupakis, Massimo Milione, Giovanni Fucà, Maria Di Bartolomeo, Filippo de Braud, Filippo Pietrantonio

**Affiliations:** ^1^ Fondazione Instituto di Ricovero e Cura Carattere Scientifico (IRCCS) Istituto Nazionale dei Tumori, Milan, Italy; ^2^ Istituto Oncologico Veneto, IRCCS, Padua, Italy; ^3^ University of Pisa, Pisa, Italy; ^4^ Humanitas Cancer Center, IRCCS, Rozzano, Italy; ^5^ University of Genoa and IRCCS Azienda Ospedaliera Universitaria (AOU) San Martino-IST, Genoa, Italy; ^6^ Fondazione Poliambulanza, Brescia, Italy; ^7^ Niguarda Cancer Center, Milan, Italy; ^8^ University of Milan, Milan, Italy; ^9^ Azienda Socio-Sanitaria Territoriale Ospedale di Cremona, Cremona, Italy; ^10^ AOU Città della Salute e della Scienza di Torino, Torino, Italy; ^11^ Ente Ospedaliero Ospedali Galliera, Genoa, Italy; ^12^ University of Messina, Messina, Italy

## Abstract

**PURPOSE:**

We assessed the prognostic/predictive role of primary tumor sidedness and uncommon alterations of anti–epidermal growth factor receptor (EGFR) primary resistance (primary resistance in *RAS* and *BRAF* wild-type metastatic colorectal cancer patients treated with anti-EGFR monoclonal antibodies [PRESSING] panel) in patients with *RAS*/*BRAF* wild-type (wt) metastatic colorectal cancer (mCRC) who were randomly assigned to panitumumab plus fluorouracil, leucovorin, and oxaliplatin (FOLFOX-4) induction followed by maintenance with panitumumab with or without fluorouracil (FU) plus leucovorin (LV); Valentino trial (ClinicalTrials.gov identifier: NCT02476045).

**PATIENTS AND METHODS:**

This prespecified retrospective analysis included 199 evaluable patients with *RAS*/*BRAF* wt. The PRESSING panel included the following: immunohistochemistry (IHC) and in situ hybridization for *HER2/MET* amplification, IHC with or without RNA sequencing for *ALK/ROS1/NTRKs/RET* fusions, next-generation sequencing for *HER2*/*PIK3CAex.20/PTEN*/*AKT1* and *RAS* mutations with low mutant allele fraction, and multiplex polymerase chain reaction for microsatellite instability. PRESSING status (any positive biomarker *v* all negative) and sidedness were correlated with overall response rate (ORR), progression-free survival (PFS), and overall survival (OS) in the study population and by treatment arm.

**RESULTS:**

Overall, left- and right-sided tumors were 85.4% and 14.6%, respectively, and PRESSING-negative and -positive tumors were 75.4% and 24.6%, respectively. At a median follow-up of 26 months, inferior outcomes were consistently observed in right- versus left-sided tumors for ORR (55.2% *v* 74.1%; *P* = .037), PFS (8.4 *v* 11.5 months; *P* = .026), and OS (2-year rate: 50.2% *v* 65.1%; *P* = .062). Similar results were observed in the PRESSING-positive versus PRESSING-negative subgroup for ORR (59.2% *v* 75.3%; *P* = .030), PFS (7.7 *v* 12.1 months; *P* < .001), and OS (2-year rate: 48.1% *v* 68.1%; *P* = .021). The PFS benefit of FU plus LV added to panitumumab maintenance, reported in the study, was independent from sidedness and PRESSING status (interaction for PFS *P* = .293 and .127, respectively). However, outcomes were extremely poor in patients who received single-agent panitumumab and had right-sided tumors (median PFS, 7.7 months; 2-year OS, 38.5%) or PRESSING-positive tumors (median PFS, 7.4 months; 2-year OS, 47.0%).

**CONCLUSION:**

The combined assessment of sidedness and molecular alterations of anti-EGFR primary resistance identified a consistent proportion of patients with *RAS*/*BRAF*–wt mCRC who had inferior benefit from initial anti-EGFR–based regimens, particularly after maintenance with single-agent anti-EGFRs.

## INTRODUCTION

The decision-making algorithm of the treatment of patients with metastatic colorectal cancer (mCRC) has deeply changed in the recent years, and it should now take into account both clinical and tumor molecular features. Since the introduction of anti–epidermal growth factor (EGFR) monoclonal antibodies, the progressive refinement of the negative selection paradigm has led to notable improvements of patients’ outcomes.^1^ All current guidelines recommend consideration of an anti-EGFR–based first-line therapy after the evaluation of *RAS* and *BRAF* mutational status in addition to assessment of primary tumor sidedness.^2,3^ Because of the negative predictive role of *RAS* and *BRAF* mutations and right sidedness, patients with left-sided, *RAS* and *BRAF* wild-type mCRC currently are regarded as optimal candidates for anti-EGFR agents alone or in combination with chemotherapy.^4-9^ However, several gaps in knowledge about primary resistance to EGFR inhibition exist, and more negative predictive biomarkers would be clinically useful in both left- and right-sided primary tumors.

In a recent case-control study in patients with *RAS* and *BRAF* wild-type mCRC treated with single-agent anti-EGFR therapy,^10^ we demonstrated the promising negative predictive impact of a panel of uncommon molecular alterations linked to primary resistance to EGFR inhibition. This panel, the Primary resistance in *RAS* and *BRAF* wild-type metastatic colorectal cancer patients treated with anti-EGFR monoclonal antibodies (PRESSING) panel, includes *HER2* amplification/activating mutations; *MET* amplification; *NTRK/ROS1/ALK/RET* rearrangements; *PIK3CA* exon 20, and *PTEN* and *AKT1* mutations.

Here, we present the results of a prespecified exploratory analysis of the Valentino study (ClinicalTrials.gov identifier: NCT02476045) to investigate the prognostic role of tumor sidedness and PRESSING panel in patients with *RAS* and *BRAF* wild-type mCRC who were randomly assigned to maintenance with either single-agent panitumumab or panitumumab plus fluorouracil and leucovorin (FU + LV) after a 4-month induction with panitumumab plus fluorouracil, leucovorin, and oxaliplatin (FOLFOX-4).

## PATIENTS AND METHODS

### Study Population

The Valentino study was a multicenter, randomized, open-label, phase II trial that investigated the progression-free survival (PFS) noninferiority of maintenance with single-agent panitumumab (arm B) versus panitumumab plus FU plus LV (arm A) after an induction treatment with panitumumab plus FOLFOX-4 in patients with *RAS* wild-type mCRC.^11^ The trial enrolled 229 patients (arm A, n = 117; arm B, n =112) and showed that maintenance with single-agent panitumumab is inferior to panitumumab plus FU/LV in terms of PFS.

The main inclusion criteria were as follows: histologically confirmed CRC with *RAS* (exons 2, 3, and 4 of both *KRAS* and *NRAS*) wild-type status confirmed by approved methods; an Eastern Cooperative Oncology Group performance score (ECOG PS) of 0 to 1; no previous treatment of metastatic disease, unresectable metastases, measurable, or just-evaluable disease according to RECIST version 1.1; and availability of baseline tumor samples centrally collected at the coordinating center (Fondazione IRCCS Istituto Nazionale dei Tumori). Patients were excluded if they had experienced relapse during adjuvant oxaliplatin-based chemotherapy or within 12 months from its completion (or within 6 months for adjuvant fluoropyrimidine monotherapy) or in case of notable comorbidities.

For this exploratory analysis, we selected all those patients enrolled in the trial with at least one radiologic disease assessment and with tumor tissue specimens obtained before enrollment and available for a complete molecular analysis, including PRESSING panel and *RAS*/*BRAF* mutational status centrally determined at the coordinating center via next-generation sequencing (NGS). Institutional review board and ethics committee approvals were obtained from all participating centers. All of the patients provided written informed consent before any study-related procedures occurred.

### Molecular Analyses

The PRESSING panel analysis included the following genomic alterations, as previously reported: *HER2* amplification/activating mutations; *MET* amplification; *NTRK/ROS1/ALK/RET* rearrangements; *PIK3CA* exon 20 mutations, *PTEN* inactivating mutations, and *AKT1* mutations.^10^ Briefly, immunohistochemistry (IHC) for HER2/MET and dual-color silver in situ hybridization for both genes were performed. IHC analyses for ALK/ROS1/panTRK/RET were performed as the screening method for actionable gene fusions; in all samples with evidence of IHC staining of any intensity/extension, whole-transcriptome shotgun sequencing (RNA-seq) was performed to confirm the presence of specific rearrangements. Oncogenic mutations in the hotspot regions of 50 cancer-related genes (Cancer Hotspot Panel v2; ThermoFisher Scientific, Waltham, MA), including *HER2* and *PIK3CA/PTEN/AKT1*, were assessed by means of targeted NGS through the Ion Torrent Personal Genome platform (ThermoFisher Scientific). In addition, by means of targeted NGS, *RAS* and *BRAF* mutational status was centrally reassessed with deeper coverage, and the fractional abundance of *BRAF* and *RAS* mutant allele fractions (MAFs) was reported after correction for tumor cellularity.^12^

On the basis of recent data on microsatellite instability (MSI) as a poor predictive factor in patients who received anti-EGFR–based first-line therapy,^13^ multiplex polymerase-chain reaction (PCR) was performed to evaluate MSI status. For additional details, see the Appendix (online only).

### Statistical Analysis

PFS was defined as the interval from random assignment to first objective documentation of progressive disease (PD) or death as a result of any cause, whichever occurred first (censored at last follow-up for patients alive and without PD). Overall survival (OS) was the interval from random assignment to death as a result of any cause (censored at last follow-up for patients alive). Overall response rate (ORR) was defined as the proportion of patients who achieved a complete (CR) or partial response (PR). Binomial two-sided 95% CIs were calculated for ORR. Survival analyses were performed using the Kaplan-Meier method and the Cox proportional-hazards model. Variables with a *P* value of < .1 at univariable analysis were entered into the multivariable models. An interaction term was included in the statistical models when subgroup analyses were performed. Median follow-up was calculated by the reverse Kaplan-Meier approach. The χ^2^ test, the Fisher exact test, or the Mann-Whitney *U* test was used, as appropriate, to evaluate the association between patient baseline characteristics and tumor sidedness or PRESSING panel status. The χ^2^ test or Fisher exact test was used, as appropriate, to assess the association between sidedness and/or PRESSING panel status with ORR. All tests were two sided at α of 5%. The analyses were carried out using R (version 3.5.0) and R Studio (version 1.1.447) and the survival, survminer, and epitools packages.

## RESULTS

### Baseline Characteristics

**FIG A1.**
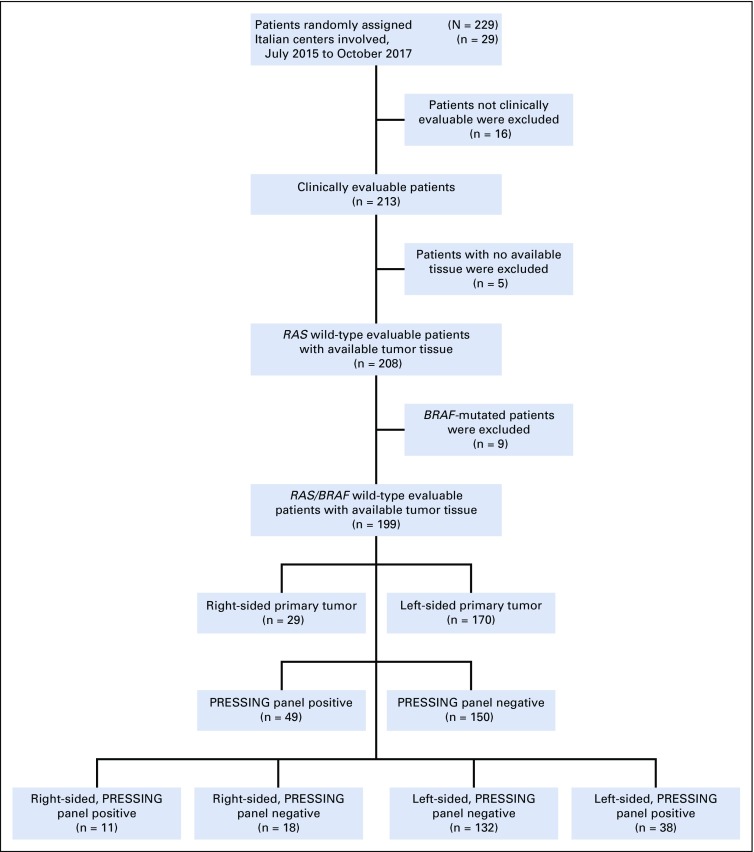
CONSORT diagram of the study.

A total of 199 (87%) of the 229 enrolled in the Valentino study were eligible for this prespecified exploratory analysis. The CONSORT diagram of the study is illustrated in Appendix Figure A1 (online only).

Baseline patients and disease characteristics are listed in Table 1. Overall, 52.3% and 47.7% patients were treated in arms A and B, respectively. Left- and right-sided tumors accounted for 170 (85.4%) and 29 (14.6%) patient cases, and the PRESSING panel was negative in 150 (75.4%) patients and positive in 49 (24.6%) patients.

**TABLE 1.**
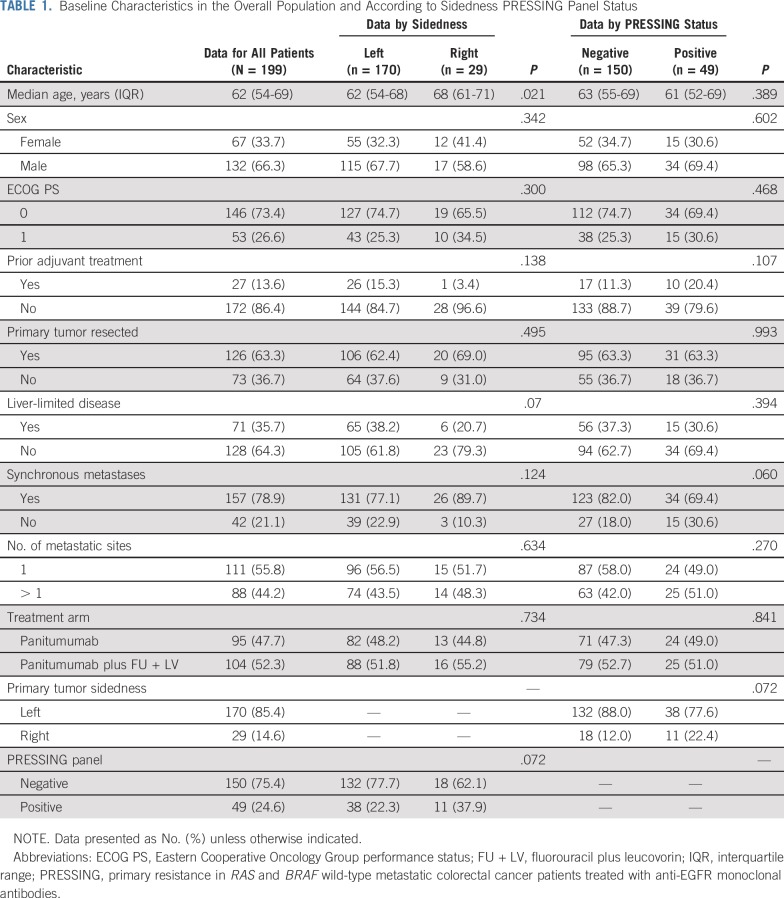
Baseline Characteristics in the Overall Population and According to Sidedness PRESSING Panel Status

**FIG 1.**
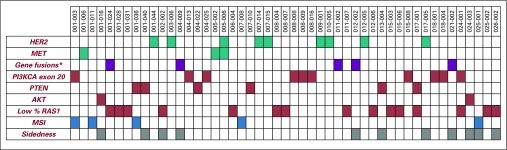
Heatmap detailing the incidence of the genomic alterations included in the primary resistance in *RAS* and *BRAF* wild-type metastatic colorectal cancer patients treated with anti-EGFR monoclonal antibodies (PRESSING) panel study population. Green indicates amplifications, violet, gene fusions, and red, mutations. Blue indicates patients with high microsatellite instability (MSI) status; gray indicates patients with right-sided tumors. (*) Targeted screening for *ALK*, *ROS1*, *NTRKs*, *RET* fusions; (†) mutant allele fraction < 5%.

The incidence of the singular molecular alterations included in the PRESSING panel is illustrated in Figure 1 and listed in Appendix Table A1 (online only). Notably, amplifications of *HER2* and *MET* genes were present in nine patients (4.5%) and three patients (1.5%), respectively. Gene fusions were reported in five patients (2.5%); specifically, three were rearrangements of *RET*, one was of *ALK*, and one was of *NTRK*. Mutations of *PI3KCA* exon 20 were found in 10 patients (5.0%); of *PTEN*, in six (3.0%); and of *AKT1*, in two (1%). RAS mutations with low MAF (< 5%) occurred in 15 patients (7.5%). Overall, MSI-high status was detected in five patients (2.5%), of whom two (40%) had disease associated with specific PRESSING alterations and only one (20%) had right sidedness.

Regarding the associations between baseline characteristics and tumor sidedness or PRESSING panel, no significant associations were observed except for older age in right-sided tumors (*P* = .02). A borderline correlation was observed between primary tumor sidedness and PRESSING panel, with a higher rate of PRESSING positivity in right-sided tumors (37.9%) versus left-sided ones (22.3%; *P* = .07; Table 1).

**FIG A2.**
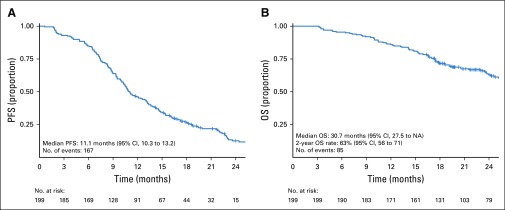
Survival analysis in the overall study population: (A) progression-free survival (PFS) and (B) overall survival (OS). NA, not assessable.

At the time of this analysis (cutoff on March 30, 2019), the median follow-up was 26 months (95% CI, 24.6 to 29 months). A total of 167 disease progressions and 85 deaths occurred. Appendix Figures A2A and A2B (online only) depict, respectively, the PFS (median, 11.1 months) and OS (median, 30.7 months; 2-year OS rate, 63%) curves in the whole-study population.

### Response Analyses According to Sidedness and PRESSING Panel

**FIG A3.**
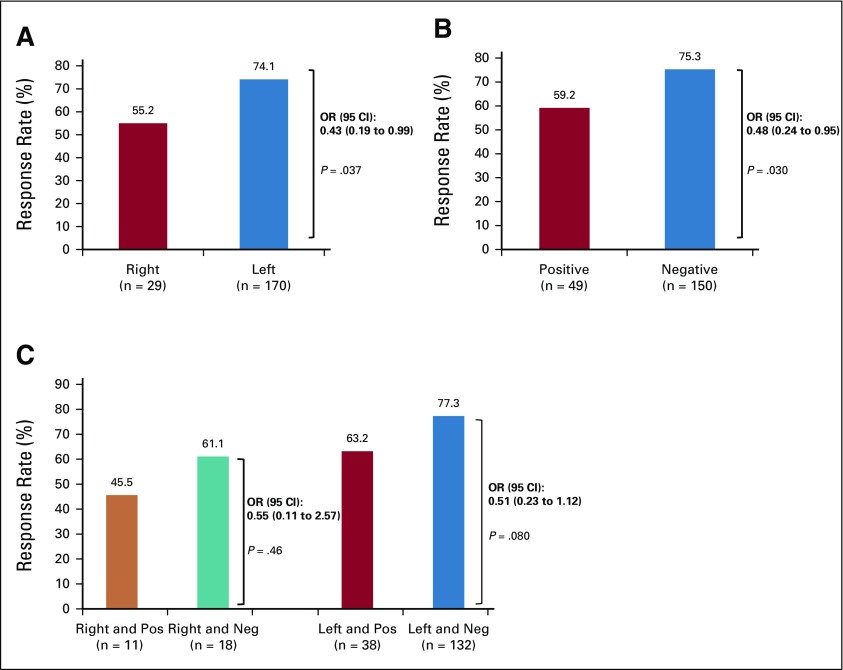
Activity analysis according to tumor sidedness and primary resistance in *RAS* and *BRAF* wild-type metastatic colorectal cancer patients treated with anti-EGFR monoclonal antibodies (PRESSING) panel status: overall response (OR) rate in patients stratified according to (A) sidedness, (B) PRESSING panel status, and (C) combined analysis. Neg, negative; Pos, positive.

**FIG A4.**
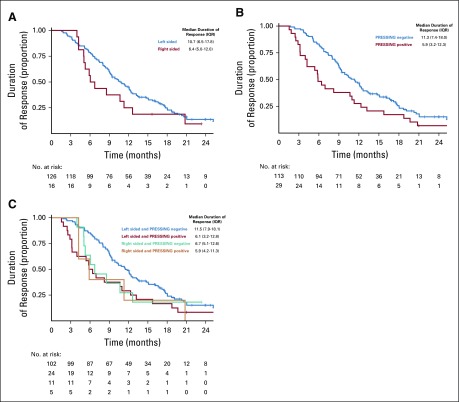
Duration of response analysis according to (A) sidedness: right sided and left sided in red and blue, respectively; (B) primary resistance in *RAS* and *BRAF* wild-type metastatic colorectal cancer patients treated with anti-EGFR monoclonal antibodies (PRESSING) panel status and (C) combined analysis. IQR, interquartile range; PRESSING, primary resistance in *RAS* and *BRAF* wild-type metastatic colorectal cancer patients treated with anti-EGFRmonoclonal antibodies.

The ORR in the study population was 75.5% (95% CI, 68.4% to 81.5%). According to sidedness, the ORR was 74.1% (95% CI, 66.9% to 80.5%) and 55.2% (95% CI, 35.7% to 73.6%) in left- and right-sided tumors, respectively (odds ratio [OR], 0.43; 95% CI, 0.19 to 0.99; *P* = .037; Appendix Fig A3A, online only). In PRESSING panel–negative and –positive tumors, the ORR was 75.3% (95% CI, 67.6% to 82.0%) and 59.2% (95% CI, 44.2% to 73.0%), respectively (OR, 0.48; 95% CI, 0.24 to 0.95; *P* = .030; Appendix Fig A3B). The ORR for patients with PRESSING-positive versus -negative tumors was 77.3% versus 63.2% (OR, 0.51; 95% CI, 0.23 to 1.12; *P* = .080) in the left-sided subgroup and was 45.6% versus 61.1% (OR, 0.55; 95% CI, 0.11 to 2.57; *P* = .466) in the right-sided subgroup (Appendix Fig A3C). Appendix Table A2 (online only) provides information on sidedness, specific PRESSING panel alterations, and RECIST response at individual patient level. Appendix Table A3 (online only) and Appendix Figure A4 (online only) summarize the results in terms of depth of response and duration of response, respectively, according to sidedness, PRESSING panel status, or both.

### Prognostic Analyses According to Sidedness and PRESSING Panel

**FIG 2.**
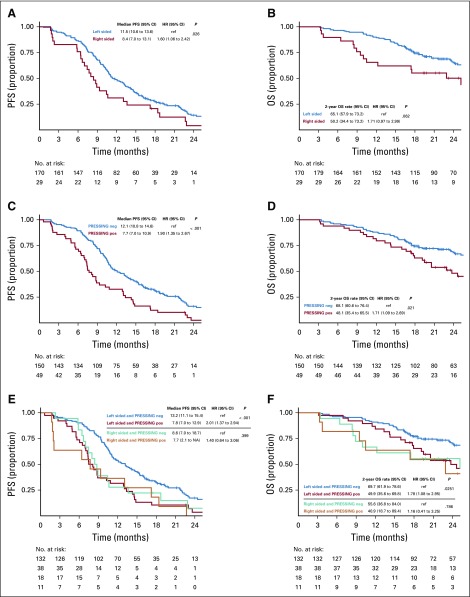
Prognostic analysis according to tumor sidedness and primary resistance in *RAS* and *BRAF* wild-type metastatic colorectal cancer patients treated with anti-EGFR monoclonal antibodies (PRESSING) panel status: Kaplan-Meier curves for (A) progression-free survival (PFS) and (B) overall survival (OS) in patients stratified according to tumor sidedness; (C) PFS and (D) OS according to PRESSING panel status; and (E) PFS and (F) OS according to the combined analysis. HR, hazard ratio; NA, not assessable; ref, reference.

**FIG A5.**
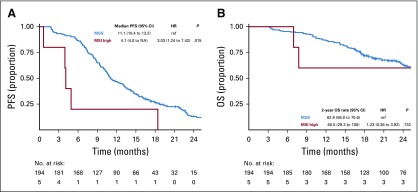
Prognostic analysis according to microsatellite instability (MSI) status: Kaplan-Meier curves for (A) progression-free survival (PFS) and (B) overall survival (OS) in patients stratified according to MSI status. HR, hazard ratio; MSS, microsatellite stable; NA, not assessable; ref, reference.

The PFS was lower in the right-sided versus left-sided subgroup (median PFS, 8.4 *v* 11.5 months; hazard ratio [HR], 1.60; 95% CI, 1.06 to 2.42; *P* = .026; Fig 2A), as was OS (2-year OS, 50.2% *v* 65.1%; HR, 1.71; 95% CI, 0.97 to 2.99; *P* = .062; Fig 2B). In parallel, PFS was lower in the PRESSING-positive versus PRESSING-negative subgroup (median PFS, 7.7 *v* 12.1 months; HR, 1.90; 95% CI, 1.35 to 2.67; *P* < .001; Fig 2C) as well as OS (2-year OS, 48.1% *v* 68.1%; HR, 1.71, 95% CI, 1.09 to 2.69; *P* = .021; Fig 2D). The median PFS of patients with PRESSING-positive versus PRESSING-negative tumors was 7.8 versus 13.2 months (HR, 2.01; 95% CI, 1.37 to 2.94; *P* < .001) in the left-sided subgroup, and it was 7.7 versus 8.6 months (HR, 1.40; 95% CI, 0.64 to 3.06; *P* = .399) in the right-sided subgroup (Fig 2E). Consistent results were observed in terms of OS: the 2-year OS of patients with PRESSING-positive versus -negative tumors was 49.9% versus 69.7% (HR, 1.78; 95% CI, 1.08 to 2.95; *P* = .025) in the left-sided subgroup and was 40.9% versus 55.6% (HR, 1.16; 95% CI, 0.41 to 3.25; *P* = .786) in the right-sided subgroup (Fig 2F). Finally, PFS was lower in the MSI-high versus microsatellite-stable subgroup (median PFS, 4.1 *v* 11.1 months; HR, 3.03; 95% CI, 1.24 to 7.42; *P* = .015; Appendix Fig A5A, online only), whereas OS was similar in the two subgroups (2-year OS, 60.0% *v* 62.9%; HR, 1.23; 95% CI, 0.38 to 3.92; *P* = .732; Appendix Fig A5B, online only).

In the univariable analysis for PFS, ECOG PS, number of metastatic sites (one *v* more than one), MSI status, primary tumor sidedness, and PRESSING panel were significantly associated with PFS; however, only ECOG PS (0 *v* 1), number of metastatic sites (one *v* more than one), and PRESSING panel confirmed their prognostic value in the multivariable model, whereas sidedness lost its significance. Similarly, in the univariable analysis for OS, ECOG PS, prior adjuvant treatment, number of metastatic sites, and PRESSING panel were significantly associated with OS; ECOG PS, prior adjuvant treatment, and PRESSING panel were confirmed in the multivariable model. In particular, the strongest association with poor PFS and OS was reported in the multivariable models for PRESSING-positive tumors (*P* < .001 and *P* = .007, respectively; Table 2).

**TABLE 2.**
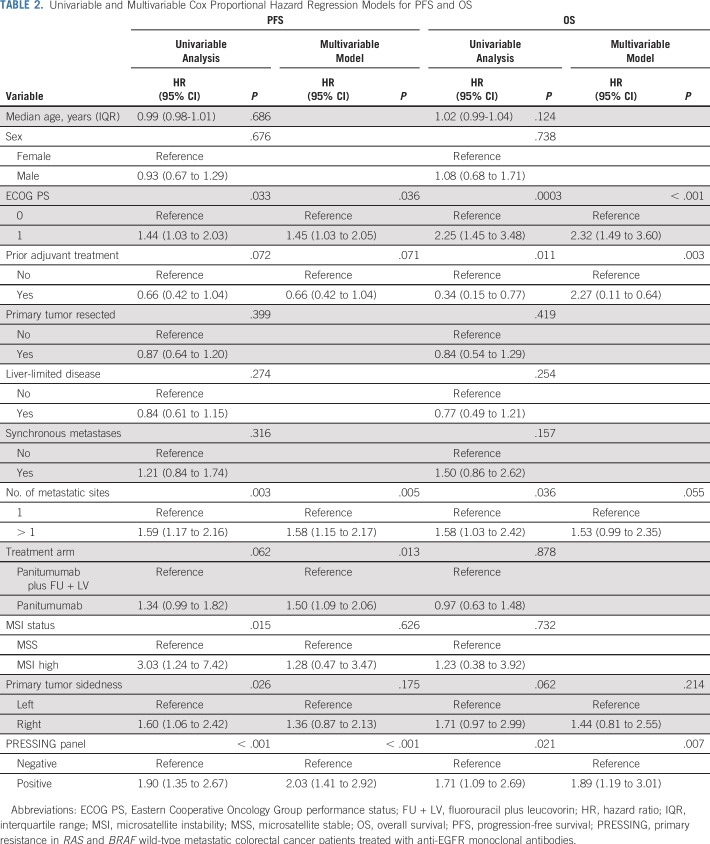
Univariable and Multivariable Cox Proportional Hazard Regression Models for PFS and OS

### Predictive Analyses According to Sidedness and PRESSING Panel

**FIG 3.**
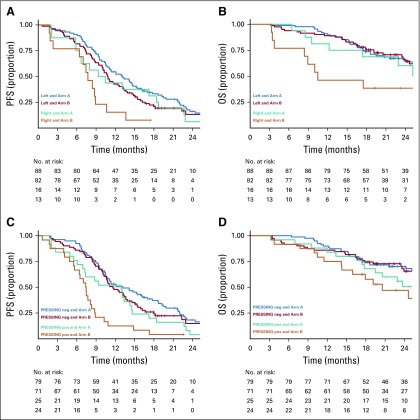
Predictive analysis according to tumor sidedness and primary resistance in *RAS* and *BRAF* wild-type metastatic colorectal cancer patients treated with anti-EGFR monoclonal antibodies (PRESSING) panel status: Kaplan-Meier curves for (A) progression-free survival (PFS) and (B) overall survival (OS) in patients stratified according to the two different maintenance treatment arms and sidedness (right- *v* left-sided tumors) and for (C) PFS and (D) OS according to treatment arm and PRESSING panel status (positive [pos] *v* negative [neg]).

**FIG A6.**
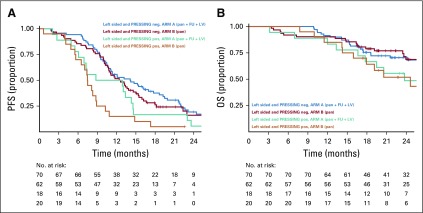
Predictive analysis according to combined tumor sidedness and primary resistance in *RAS* and *BRAF* wild-type metastatic colorectal cancer patients treated with anti-EGFR monoclonal antibodies (PRESSING) panel status in left-sided tumors. Kaplan-Meier curves for (A) progression-free survival (PFS) and (B) overall survival (OS) in the patient subgroup with left-sided/PRESSING-negative tumors stratified according to the two different maintenance treatment arms or the patient subgroup with left-sided/PRESSING-positive tumors stratified according to the two different maintenance treatment arms. FU + LV, fluorouracil plus leucovorin; neg, negative; pan, panitumumab; pos, positive.

Results about the predictive role of sidedness, PRESSING panel status, or both according to the two treatment arms are summarized in Table 3. Primary tumor sidedness was not significantly associated with differential effect of the two maintenance arms in terms of PFS and OS (*P* for interaction = .293 and .068, respectively), although the PFS and OS benefits from maintenance treatment with panitumumab plus FU plus LV were higher among patients with right- than with left-sided tumors (Figs 3A and 3B). Similar results were observed with regard to the predictive effect of the PRESSING panel for both PFS and OS (*P* for interaction = .127 and .450, respectively), although the PFS benefit from addition of FU plus LV to panitumumab in the maintenance setting was clearly superior in PRESSING-positive tumors (Figs 3C and 3D). Consistent results were found when the predictive role of the PRESSING panel was analyzed with regard to maintenance treatment arm in the subgroup of patients with left-sided tumors (Appendix Table A4, online only; Appendix Fig A6, online only), whereas the sample size was too limited to perform such analyses in the subgroup of patients with right-sided tumors.

**TABLE 3.**
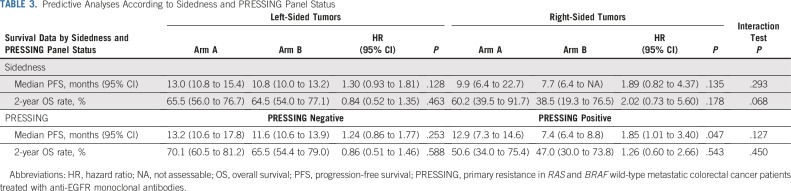
Predictive Analyses According to Sidedness and PRESSING Panel Status

## DISCUSSION

In a previous prospective, case-control study, we showed the potential negative predictive role of the PRESSING panel, including several genomic alterations selected on the basis of the most robust and biologically sound biomarkers of primary resistance to anti-EGFRs beyond *RAS* and *BRAF* mutational status and primary tumor sidedness.^10^ In this prespecified exploratory analysis of the Valentino study, we investigated the potential prognostic and predictive role of primary tumor sidedness and PRESSING panel in patients with *RAS* and *BRAF* wild-type mCRC who were randomly assigned to panitumumab plus FOLFOX-4 followed by maintenance with either single-agent panitumumab or panitumumab plus FU plus LV. We reported that a negative hyperselection beyond *RAS* and *BRAF*, obtained through the accurate analysis of multiple and less frequent genomic alterations included in the PRESSING panel, combined with the evaluation of tumor sidedness, allowed better prediction of the outcomes in this study population. In particular, patients with left-sided and PRESSING-negative tumors achieved clearly better outcomes in terms of both PFS and OS, and FU plus LV–based maintenance treatment had a positive PFS impact also in this patient subgroup.

Of note, no significant associations between baseline characteristics and tumor sidedness or PRESSING panel were observed (except for older age in right-sided tumors). As expected, the association between sidedness and PRESSING panel positivity was due to the enrichment of resistance alterations (except *HER2* amplification) in right-sided tumors.^10,14-16^ This correlation may have failed to achieve statistical significance because of the low number of right-sided tumors in the study population. However, even if primary tumor sidedness may be a surrogate marker for the heterogeneous molecular profile of mCRC, primary resistance to anti-EGFRs displayed by right-sided cancers is not fully explained by the well-known and biologically validated genomic alterations included in the PRESSING panel and may be linked to specific gene expression profiles or miRNAs, such as miR-31-3p.^17,18^

The results of this study were internally consistent, because ORR, PFS, and OS were all decreased in right-sided tumors compared with left-sided ones and in PRESSING-positive with respect to -negative ones. In the multivariable model, the PRESSING panel was the strongest prognostic factor not only in terms of PFS but also with regard to OS. Conversely, sidedness was no longer significant, again possibly because of the low number of right-sided tumors. Consistent with the literature,^13,19^ MSI-high status was associated with poor PFS outcome at the univariable analysis, although the number of MSI-high occurrences in this data set was quite small (only five patients) and did not allow us to properly assess its independent prognostic role. The type of maintenance treatment retained its value in terms of PFS but not OS; this result has been already reported^11^ and may be due to the low number of OS events at the time of data cutoff and to the underpowered sample size.

Interestingly, we observed that ORR and depth of response were numerically increased in patients with right-sided/PRESSING-negative versus right-sided/PRESSING-positive tumors. A similar role of the PRESSING panel was observed in the left-sided subgroup. However, in the specific subgroup of patients with right-sided tumors, the increase of response rate achieved thanks to negative hyperselection failed to translate into a benefit in terms of duration of response, PFS, or OS. This is in line with post hoc analyses of pivotal trials and meta-analyses that investigated the impact of sidedness on ORR versus survival end points in patients with *RAS* wild-type mCRC who received anti-EGFR–based treatment.^8,20,21^ On the basis of such results, an anti-EGFR–based first-line treatment rarely may be offered on an individual basis to patients with right-sided *RAS* wild-type mCRC, at least whenever tumor response is the primary goal of treatment and particularly when antiangiogenics and/or triplet chemotherapy are contraindicated. Given the higher prevalence of PRESSING panel alterations in right-sided tumors, the role of negative molecular hyperselection may be crucial for some patients with *RAS* and *BRAF* wild-type/right-sided tumors.

Furthermore, the PFS benefit of FU plus LV added to panitumumab in the maintenance setting was independent from sidedness and PRESSING panel status, which thus confirmed the crucial role of fluoropyrimidine continuation in the maintenance setting. However, PFS was extremely poor in patients with right-sided or PRESSING-positive disease treated with single-agent panitumumab, with an abrupt decrease of the curves after 4 months (which corresponded to the end of the induction phase). This result highlights that single-agent anti-EGFRs should not be regarded as an effective maintenance treatment strategy in disease subgroups with a lack of clinically or molecularly defined EGFR dependency. In these subgroups, de-escalation to a fully chemotherapy-free maintenance strategy was associated with a significant loss of efficacy, and, in patients with right-sided mCRC (which is itself associated with poorer outcomes^22^), a detrimental effect was observed even in terms of OS.

This study has some clear limitations. First of all, because both maintenance treatment arms contained panitumumab, we could not investigate the predictive role of tumor sidedness and PRESSING panel status with regard to anti-EGFR therapy. However, because FU plus LV was administered only in arm A, we could identify a subgroup of patients (ie, right-sided and/or PRESSING-positive disease) who derived a limited benefit from single-agent panitumumab, which confirmed the fundamental role of chemotherapy for maintenance treatment and suggested the limited clinical benefit from anti-EGFR treatment itself in these subsets. Most important, the results of this study should be interpreted with caution, because the role of anti-EGFR therapy added to FU/LV in the maintenance setting is still not established by level I evidence. This is particularly relevant in light of the current lack of comparison with other evidence-based maintenance options that have better long-term tolerability, such as FU plus LV with or without bevacizumab. Ongoing studies, such as Panama (ClinicalTrials.gov identifier: NCT01991873) and FIRE-4 (ClinicalTrials.gov identifier: NCT02934529), we hope will address the still-unanswered questions with their adequate randomized settings.

Also, we acknowledge that, although tumor sidedness is a simple, clearly definable and homogeneous variable, the PRESSING panel is a composite biomarker that includes several genomic alterations. Therefore, each genomic alteration may constitute a single marker, endowed with a potential differential prognostic and/or predictive effect, and our results do not distinguish the relative contribution of individual variables because of the extremely low prevalence of each. The complex molecular interactions of these candidate genomic alterations in the neoplastic signaling pathways and their low prevalences limit their formal validation in prospective clinical studies or in post hoc analyses of randomized clinical trials as negative predictive markers for response to EGFR-targeted therapies, and this limitation may impair their implementation in the tumor profiling work-up, even if many of them are validated therapeutic targets.^10,14-16,23-29^ Regarding *RAS* mutations with an MAF less than the 5% cutoff, which was validated for negative selection of patients for anti-EGFRs, it is still unclear whether mutations with low fractional abundance simply mirror tumor heterogeneity that may be overcome by novel techniques, such as liquid biopsy,^30^ or may be associated with the rapid onset of acquired resistance and limited long-term PFS benefit under the selective pressure of anti-EGFR agents continued until disease progression develops.^31^

In conclusion, even if patients with left-sided, *RAS* and *BRAF* wild-type tumors currently are considered the optimal candidates for EGFR inhibitors,^3^ a consistent proportion of them achieve a significantly inferior clinical benefit from upfront anti-EGFR–based regimens, particularly after de-escalation to maintenance treatment with single-agent anti-EGFRs. A negative molecular hyperselection with our PRESSING panel, added to the initial assessment of sidedness and *RAS/BRAF* mutational status, may help identify a subgroup of patients who will exceptionally benefit from anti-EGFR–based initial therapy.

## Figures and Tables

**TABLE A1.**
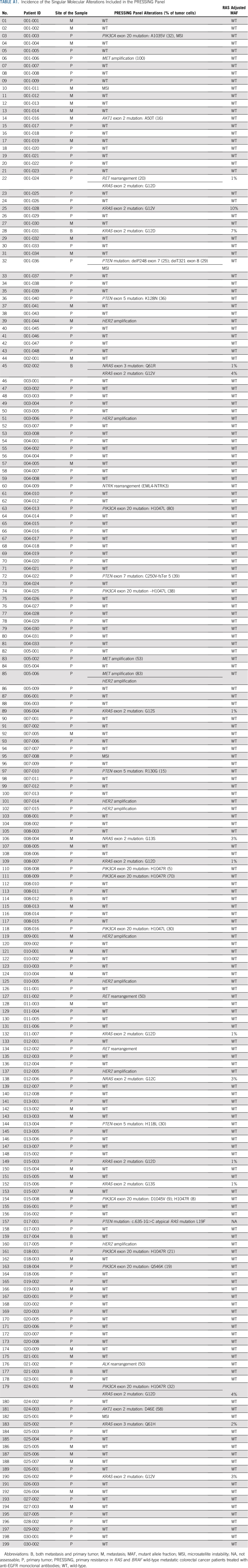
Incidence of the Singular Molecular Alterations Included in the PRESSING Panel

**TABLE A2.**
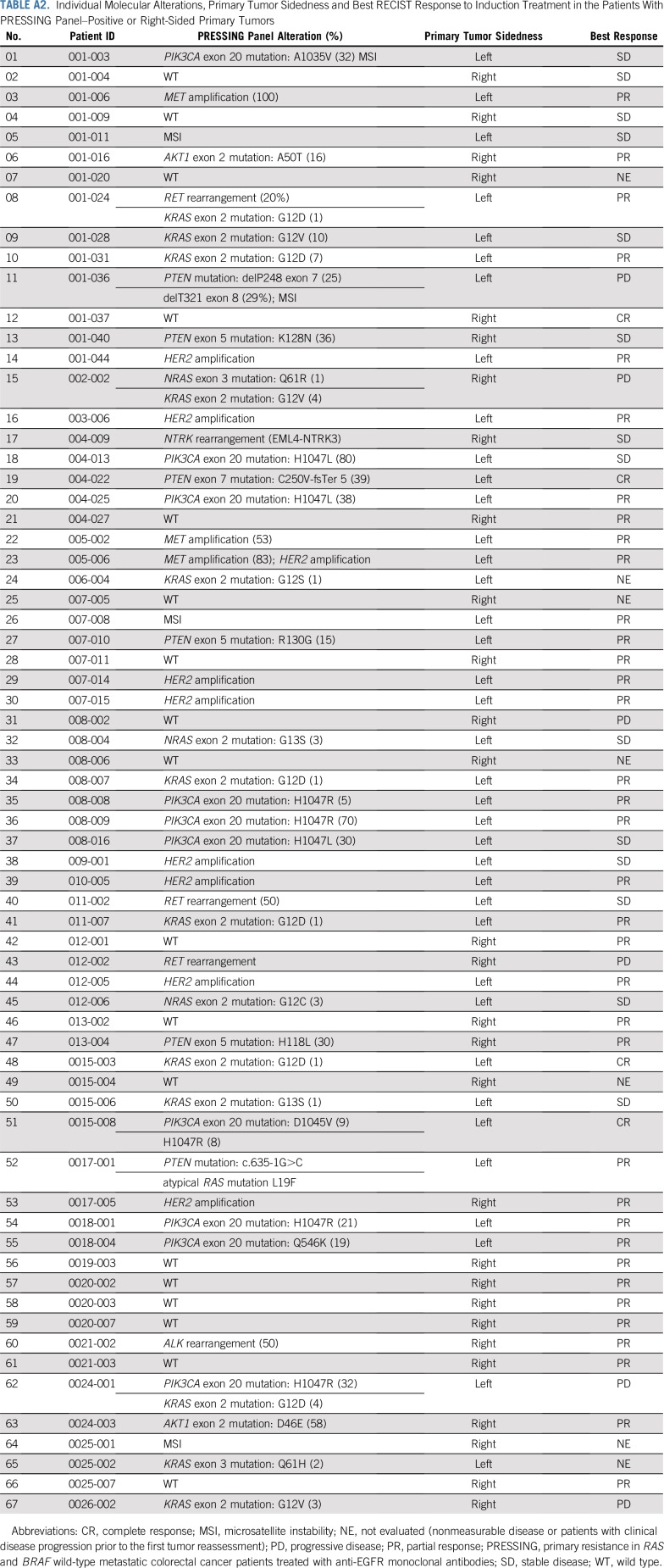
Individual Molecular Alterations, Primary Tumor Sidedness and Best RECIST Response to Induction Treatment in the Patients With PRESSING Panel–Positive or Right-Sided Primary Tumors

**TABLE A3.**
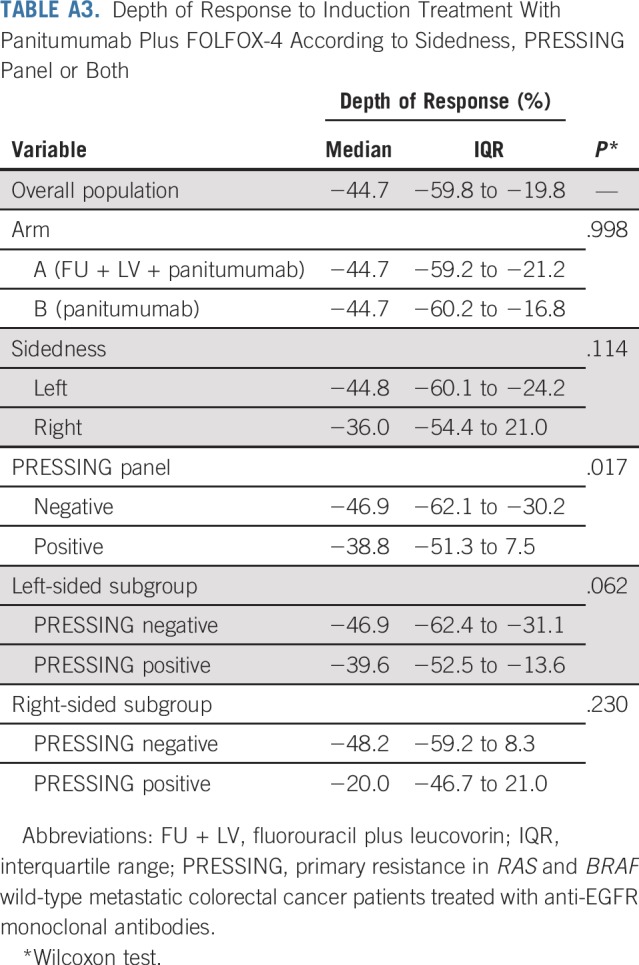
Depth of Response to Induction Treatment With Panitumumab Plus FOLFOX-4 According to Sidedness, PRESSING Panel or Both

**TABLE A4.**
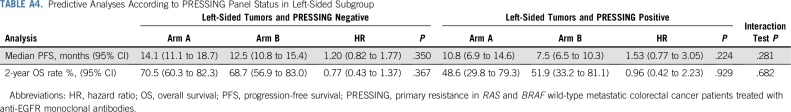
Predictive Analyses According to PRESSING Panel Status in Left-Sided Subgroup

## Appendix

### Supplementary Methods

The primary resistance in *RAS* and *BRAF* wild-type metastatic colorectal cancer patients treated with anti-EGFR monoclonal antibodies (PRESSING) panel analysis was performed as previously described.^10^ Specifically, immunohistochemistry (IHC) for *HER2*/*MET* and dual-color silver in situ hybridization (SISH) for both genes were carried out and scored as described previously (Pietrantonio F, et al: Clin Cancer Res 23: 2412-2422, 2017; Valtorta E, et al: Mod Pathol 28:1481-1491, 2015). In detail, IHC was performed on 3-μm formalin-fixed paraffin-embedded (FFPE) tissue sections or on WiDr cytoclots. *MET* protein expression was detected by a rabbit monoclonal anti-MET antibody (dilution 1:200; clone SP44; Spring Bioscience, Pleasanton, CA) directed against the synthetic peptide derived from the C terminus of human MET that displayed membranous and/or cytoplasmic epitope. *HER2* expression analysis was performed using the HercepTest antibody (Agilent; Santa Clara, CA) and automatically on the automated Benchmark Ultrasystem (Ventana Medical Systems, Tucson, AZ) using the Ventana 4B5 antibody according to the manufacturers’ instructions. Bright-field dual-color SISH analysis was performed on 3-μm FFPE tissue sections using the MET DNP Probe (Ventana Medical Systems) along with the Chromosome 7 DIG Probe (Ventana Medical Systems) on a BenchMark Ultra Platform (Ventana Medical Systems) according to the manufacturer’s protocol. *HER2* amplification analysis by SISH with a Ventana Medical Systems 4B5 Inform HER2 dual color on the BenchMark Ultra system (Inform HER2 DNA dual-color assay; Roche Tissue Diagnostics, Ventana Medical Systems). The scoring and evaluation for in situ hybridization was performed by counting *HER2* and *CEN17* signals from 100 nuclei per case. Nontumor tissue (normal colon mucosa) was used as an internal negative control. *HER2* gene amplification was defined as positive when the *HER2/CEP17* ratio was two or greater or the average number of *HER2* signals per tumor cell nucleus was more than 6, whereas *MET* amplification was defined as positive when the *MET/CEP7* ratio was two or greater or average number of *MET* signals per tumor cell nucleus was more than 6.

IHC for ALK/ROS1/panTRK/RET was carried out as screening method using standard protocols for pan-Trk (including TrkA, TrkB, TrkC; Cell Signaling, Danvers, MA; clone C17F1, 1:25 dilution), ROS1 (Cell Signaling; clone D4D6, 1:500 dilution), ALK (Cell Signaling, Danvers, MA; clone D5F3, 1:500 dilution) and RET (Abcam, Cambridge, United Kingdom; clone EPR2871). In all samples with evidence of IHC staining of any intensity/extension, whole-transcriptome shotgun sequencing (RNA-seq) was performed to confirm the presence of specific rearrangements and to identify the specific fusion partner.^14^

Mutational analysis was performed on FFPE specimens for each case; these were sliced in 5-μm sections and manually microdissected to isolate the tumor area that carried the highest percentage of neoplastic cells—identified by a pathologist on hematoxylin and eosin. A minimal tumor percentage of 10% was required; the average tumor percentage was 70% (range, 10% to 90%), and no difference of percentages was observed between sensitive and resistant samples. Samples were treated with xylene and 100% ethanol to remove paraffin, and then DNA was isolated using the GeneRead DNA FFPE kit (catalog No. 180134; Qiagen, Hilden, Germany; ). DNA amount and quality were identified using Nano Drop platform and Qbit dsDNA BR kit (ThermoFisher Scientific, Waltham, MA) according to the manufacturer’s instructions. Oncogenic mutations in the hotspot regions of 50 oncogenes and tumor suppressor genes commonly mutated in human cancers (Cancer Hotspot Panel v2, ThermoFisher Scientific: *ABL1*, *AKT1*, *ALK*, *APC*, *ATM*, *BRAF*, *CDH1*, *CDKN2A*, *CSF1R*, *CTNNB1*, *EGFR*, *ERBB2*, *ERBB4*, *EZH2*, *FBXW7*, *FGFR1*, *FGFR2*, *FGFR3*, FLT3, *GNA11*, *GNAS*, *GNAQ*, *HNF1A*, *HRAS*, *IDH1*, *IDH2*, *JAK2*, *JAK3*, *KDR*/*VEGFR2*, *KIT*, *KRAS*, *MET*, *MLH1*, *MPL*, *NOTCH1*, *NPM1*, *NRAS*, *PDGFRA*, *PIK3CA*, *PTEN*, *PTPN11*, *RB1*, *RET*, *SMAD4*, *SMARCB1*, *SMO*, *SRC*, *STK11*, *TP53*, *VHL*) were assessed by means of targeted next-generation sequencing through the Ion Torrent Personal Genome platform (ThermoFisher Scientific) , according to the manufacturer’s instructions (Peitrantonio F, et al: Ann Oncol 27:2097-2103, 2016; Pietrantonio F, et al: Clin Cancer Res 24:1082-1089, 2018).

The *BRAF* and *RAS* mutational status was centrally reassessed through targeted next-generation sequencing with deeper coverage to detect low-percentage and atypical *RAS* mutations. The fractional abundances of *BRAF* and *RAS* mutations, called mutant allele fractions (MAFs) were reported. Average sequencing depth was 1,000×, and mutations were defined with a minimum MAF of 3%. MAF was corrected for tumor cellularity, defined as the percentage of tissue sample occupied by tumor cells on the total amount of cells, including stromal microenvironment and inflammatory infiltrate.^12^

Microsatellite instability status (MSI) analysis was performed after DNA was extracted from each tumor block and amplified via polymerase chain reaction. The MSI status was identified using five quasi-monomorphic mononucleotide markers able to provide highly accurate determinations of the tumor MSI status from DNA: BAT-25, BAT-26, NR-21, NR-24, and MONO-27 (MSI Analysis System, version 1.2; Promega, Madison, WI). According to previous evidence, cases with instability at two or more of the five markers were classified as MSI high, whereas samples with instability at one marker and without instability were categorized as MSI low and microsatellite stable, respectively (Smyth EC, et al: JAMA Oncol 3:1197-1203, 2017).

## References

[1] Van Cutsem E Lenz HJ Köhne CH et al Fluorouracil, leucovorin, and irinotecan plus cetuximab treatment and *RAS* mutations in colorectal cancer J Clin Oncol 33 692 700 2015 25605843 10.1200/JCO.2014.59.4812

[2] Van Cutsem E Cervantes A Adam R et al ESMO consensus guidelines for the management of patients with metastatic colorectal cancer Ann Oncol 27 1386 1422 2016 27380959 10.1093/annonc/mdw235

[3] National Comprehensive Cancer Network Colon cancer (version 1.2019). https://www.nccn.org/professionals/physician_gls/pdf/colon.pdf

[4] Van Cutsem E Köhne CH Láng I et al Cetuximab plus irinotecan, fluorouracil, and leucovorin as first-line treatment for metastatic colorectal cancer: Updated analysis of overall survival according to tumor *KRAS* and *BRAF* mutation status J Clin Oncol 29 2011 2019 2011 21502544 10.1200/JCO.2010.33.5091

[5] Venook AP Niedzwiecki D Lenz HJ et al Effect of first-line chemotherapy combined with cetuximab or bevacizumab on overall survival in patients with *KRAS* wild-type advanced or metastatic colorectal cancer: A randomized clinical trial JAMA 317 2392 2401 2017 28632865 10.1001/jama.2017.7105 PMC5545896

[6] Heinemann V von Weikersthal LF Decker T et al FOLFIRI plus cetuximab versus FOLFIRI plus bevacizumab as first-line treatment for patients with metastatic colorectal cancer (FIRE-3): A randomised, open-label, phase 3 trial Lancet Oncol 15 1065 1075 2014 25088940 10.1016/S1470-2045(14)70330-4

[7] Douillard JY Oliner KS Siena S et al Panitumumab-FOLFOX4 treatment and *RAS* mutations in colorectal cancer N Engl J Med 369 1023 1034 2013 24024839 10.1056/NEJMoa1305275

[8] Arnold D Lueza B Douillard JY et al Prognostic and predictive value of primary tumour side in patients with *RAS* wild-type metastatic colorectal cancer treated with chemotherapy and *EGFR* directed antibodies in six randomized trials Ann Oncol 28 1713 1729 2017 28407110 10.1093/annonc/mdx175 PMC6246616

[9] Holch JW Ricard I Stintzing S et al The relevance of primary tumour location in patients with metastatic colorectal cancer: A meta-analysis of first-line clinical trials Eur J Cancer 70 87 98 2017 27907852 10.1016/j.ejca.2016.10.007

[10] Cremolini C Morano F Moretto R et al Negative hyper-selection of metastatic colorectal cancer patients for anti-EGFR monoclonal antibodies: The PRESSING case-control study Ann Oncol 28 3009 3014 2017 29045518 10.1093/annonc/mdx546

[11] Pietrantonio F Morano F Corallo S et al Maintenance therapy with panitumumab alone vs panitumumab plus fluorouracil-leucovorin in patients with *RAS* wild-type metastatic colorectal cancer: A phase 2 randomized clinical trial JAMA Oncoldoi:10.1001/jamaoncol.2019.1467 [epub ahead of print on July 3, 2019] 10.1001/jamaoncol.2019.1467 PMC6613306 31268481

[12] Dienstmann R Elez E Argiles G et al Analysis of mutant allele fractions in driver genes in colorectal cancer: Biological and clinical insights Mol Oncol 11 1263 1272 2017 28618197 10.1002/1878-0261.12099 PMC5579330

[13] Innocenti F Ou FS Qu X et al Mutational analysis of patients with colorectal cancer in CALGB/SWOG 80405 identifies new roles of microsatellite instability and tumor mutational burden for patient outcome J Clin Oncol 37 1217 1227 2019 30865548 10.1200/JCO.18.01798 PMC6506418

[14] Pietrantonio F Di Nicolantonio F Schrock AB et al *ALK*, *ROS1*, and *NTRK* rearrangements in metastatic colorectal cancer J Natl Cancer Inst 109 djx089 2017 10.1093/jnci/djx089 29370427

[15] Pietrantonio F Di Nicolantonio F Schrock AB et al *RET* fusions in a small subset of advanced colorectal cancers at risk of being neglected Ann Oncol 29 1394 1401 2018 29538669 10.1093/annonc/mdy090

[16] Sartore-Bianchi A Trusolino L Martino C et al Dual-targeted therapy with trastuzumab and lapatinib in treatment-refractory, *KRAS* codon 12/13 wild-type, HER2-positive metastatic colorectal cancer (HERACLES): A proof-of-concept, multicentre, open-label, phase 2 trial Lancet Oncol 17 738 746 2016 27108243 10.1016/S1470-2045(16)00150-9

[17] Guinney J Dienstmann R Wang X et al The consensus molecular subtypes of colorectal cancer Nat Med 21 1350 1356 2015 26457759 10.1038/nm.3967 PMC4636487

[18] Laurent-Puig P Grisoni ML Heinemann V et al Validation of miR-31-3p expression to predict cetuximab efficacy when used as first-line treatment in *RAS* wild-type metastatic colorectal cancer Clin Cancer Res 25 134 141 2019 30108104 10.1158/1078-0432.CCR-18-1324

[19] Lenz H-J Ou F-S Venook AP et al Impact of consensus molecular subtype on survival in patients with metastatic colorectal cancer: Results from CALGB/SWOG 80405 (alliance) J Clin Oncol 37 1876 1885 2019 31042420 10.1200/JCO.18.02258 PMC6675593

[20] Tejpar S Stintzing S Ciardiello F et al Prognostic and predictive relevance of primary tumor location in patients with *RAS* wild-type metastatic colorectal cancer: Retrospective analyses of the CRYSTAL and FIRE-3 trials JAMA Oncol 3 194 201 2017 27722750 10.1001/jamaoncol.2016.3797 PMC7505121

[21] Qin S Li J Wang L et al Efficacy and tolerability of first-line cetuximab plus leucovorin, fluorouracil, and oxaliplatin (FOLFOX-4) versus FOLFOX-4 in patients with *RAS* wild-type metastatic colorectal cancer: The open-label, randomized, phase III TAILOR trial J Clin Oncol 36 3031 3039 2018 30199311 10.1200/JCO.2018.78.3183 PMC6324088

[22] Petrelli F Tomasello G Borgonovo K et al Prognostic survival associated with left-sided vs right-sided colon cancer: A systematic review and meta-analysis JAMA Oncol 3 211 219 2017 27787550 10.1001/jamaoncol.2016.4227

[23] De Roock W Claes B Bernasconi D et al Effects of *KRAS*, *BRAF*, *NRAS*, and *PIK3CA* mutations on the efficacy of cetuximab plus chemotherapy in chemotherapy-refractory metastatic colorectal cancer: A retrospective consortium analysis Lancet Oncol 11 753 762 2010 20619739 10.1016/S1470-2045(10)70130-3

[24] Yonesaka K Zejnullahu K Okamoto I et al Activation of *ERBB2* signaling causes resistance to the EGFR-directed therapeutic antibody cetuximab Sci Transl Med 3 99ra86 2011 10.1126/scitranslmed.3002442 PMC3268675 21900593

[25] Bardelli A Corso S Bertotti A et al Amplification of the MET receptor drives resistance to anti-EGFR therapies in colorectal cancer Cancer Discov 3 658 673 2013 23729478 10.1158/2159-8290.CD-12-0558 PMC4078408

[26] Perrone F Lampis A Orsenigo M et al *PI3KCA*/*PTEN* deregulation contributes to impaired responses to cetuximab in metastatic colorectal cancer patients Ann Oncol 20 84 90 2009 18669866 10.1093/annonc/mdn541

[27] Mao C Liao RY Chen Q Loss of *PTEN* expression predicts resistance to EGFR-targeted monoclonal antibodies in patients with metastatic colorectal cancer Br J Cancer 102 940 2010 20160728 10.1038/sj.bjc.6605575 PMC2833261

[28] Overman MJ McDermott R Leach JL et al Nivolumab in patients with metastatic DNA mismatch repair-deficient or microsatellite instability-high colorectal cancer (CheckMate 142): An open-label, multicentre, phase 2 study Lancet Oncol 18 1182 1191 2017 28734759 10.1016/S1470-2045(17)30422-9 PMC6207072

[29] Overman MJ Lonardi S Wong KYM et al Durable clinical benefit with nivolumab plus ipilimumab in DNA mismatch repair-deficient/microsatellite instability-high metastatic colorectal cancer J Clin Oncol 36 773 779 2018 29355075 10.1200/JCO.2017.76.9901

[30] Antoniotti C Pietrantonio F Corallo S et al Circulating tumor DNA analysis in colorectal cancer: From dream to reality JCO Precis Oncol 10.1200/PO.18.00397 10.1200/PO.18.00397 35100685

[31] Van Emburgh BO Arena S Siravegna G et al Acquired *RAS* or *EGFR* mutations and duration of response to EGFR blockade in colorectal cancer Nat Commun 7 13665 2016 27929064 10.1038/ncomms13665 PMC5155160

